# Ovarian Epithelioid Hemangioendothelioma in a Pediatric Patient: A Case Report

**DOI:** 10.7759/cureus.22556

**Published:** 2022-02-24

**Authors:** Brian D Noreña-Rengifo, Mónica Royero-Arias, Antonella Arrieta-Rojano, Jorge Ochoa-Gaviria, Abraham Chams-Anturi

**Affiliations:** 1 Radiology, Universidad de Antioquia, Medellín, COL; 2 Pediatric Radiology, Servicios de Salud San Vicente Fundación, Medellín, COL; 3 Pediatric Radiology, Hospital Infantil San Vicente Fundación, Medellín, COL; 4 Pediatric Radiology, Hospital Pablo Tobón Uribe, Medellín, COL; 5 Pediatric Surgery, Hospital San Vicente Fundación, Medellín, COL

**Keywords:** pediatric, epithelioid hemangioendothelioma, ovaries, vascular tumors, childhood, ovarian neoplasm, mri pelvis, pelvic ultrasonography

## Abstract

We present a case of an eight-month-old girl who was brought to the emergency department with bloody stools. An initial ultrasound reported a mass in the left iliac fossa that was further characterized by magnetic resonance imaging (MRI) as a hypervascular ovarian tumor. Prior to surgical resection of the tumor, abdominal arteriography with selective embolization and vessel occlusion was performed. Pathology reported epithelioid hemangioendothelioma of the left ovary. This condition has not been previously reported in girls. In this case report, we describe the ultrasound, MRI, and arteriographic findings with a histopathologic correlation of an adnexal tumor that is unknown in the pediatric female population until now.

## Introduction

Epithelioid hemangioendothelioma (EHE) is a rare vascular tumor, originating from endothelial or pre-endothelial vascular cells [1] and mainly affecting young women [2]. It is generally asymptomatic and is diagnosed incidentally. It is multicentric, with lung, liver, bone, and soft tissue involvement, and rarely in the genitals [1,3]. The clinical course and prognosis are unpredictable. Due to its low prevalence, there is no standard treatment and a poor response to chemotherapy is common [1,4,5]. In this case report, we present the imaging characteristics of an ovarian EHE in a pediatric patient for the first time in the literature.

## Case presentation

**Figure 1:**
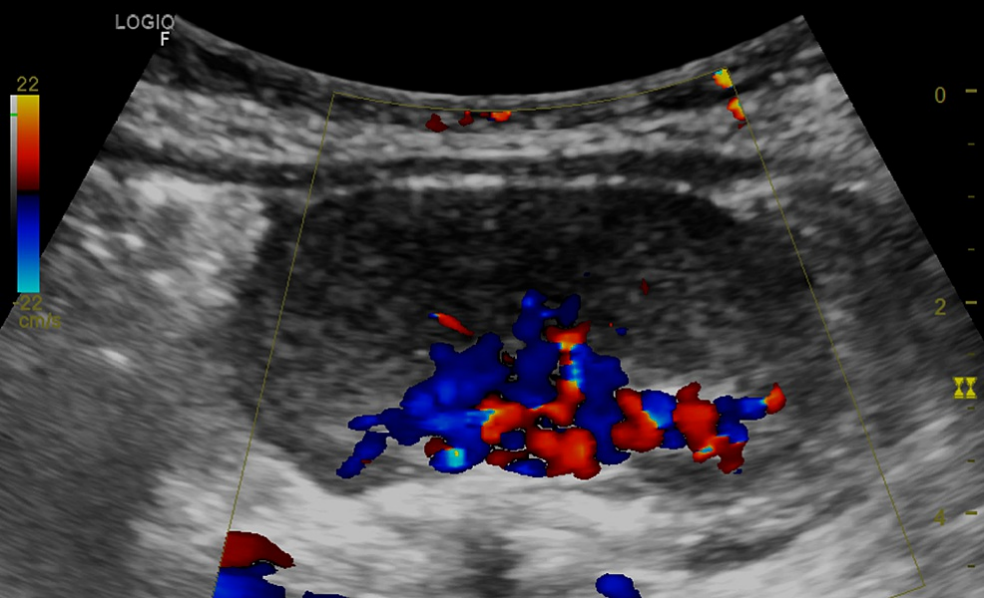
Abdominal ultrasound. A solid lesion in the left iliac fossa with heterogeneous echogenicity and significant arterial and venous vascularization in color Doppler mode.

An eight-month-old girl patient with no personal medical history was transferred to the emergency department of a highly complex children's hospital in Medellín, Colombia, due to fever and “red currant jelly” stools. Initially, an ultrasound was performed, where a solid mass was observed in the left iliac fossa with heterogeneous echogenicity, without calcifications, and with significant central arterial and venous vascularization in color Doppler mode (Figure 1).

**Figure 2:**
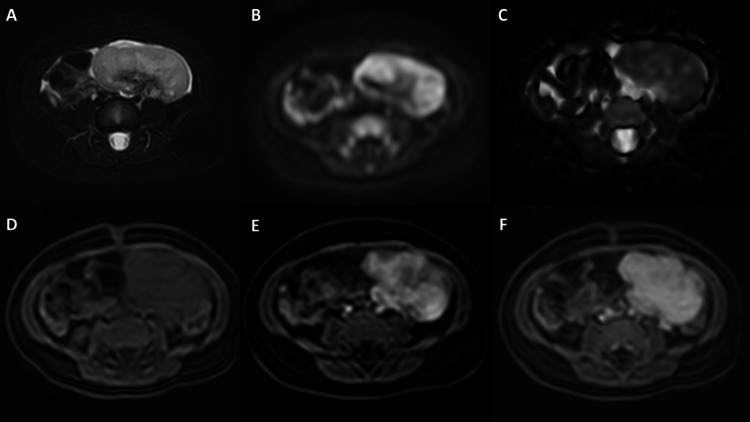
Abdominal and pelvic contrast MRI. Tumor originating in the left ovary, hyperintense on T2-weighted images (A), with a marked restriction on diffusion-weighted images (B) and the respective apparent diffusion coefficient (ADC) map (C). The tumor is isointense on T1-weighted images before gadolinium injection (D) and had intense and progressive enhancement in arterial (E) and venous (F) phases.

A magnetic resonance imaging with intravenous contrast was performed (Figure 2), identifying a solid tumor with well-defined edges, a hyperintense signal on T2, and a hypointense signal on T1, with avid and homogeneous contrast enhancement in the arterial phase that persisted in late phases. In diffusion-weighted images, the mass showed tissue restriction. The lesion measured 4 x 3.5 x 5.3 cm and had contact with the left superolateral wall of the bladder. A central vascular pedicle with irrigation from the internal iliac artery and drainage to the left gonadal vein was observed, suggesting a left adnexal origin. The right ovary appeared normal.

**Figure 3:**
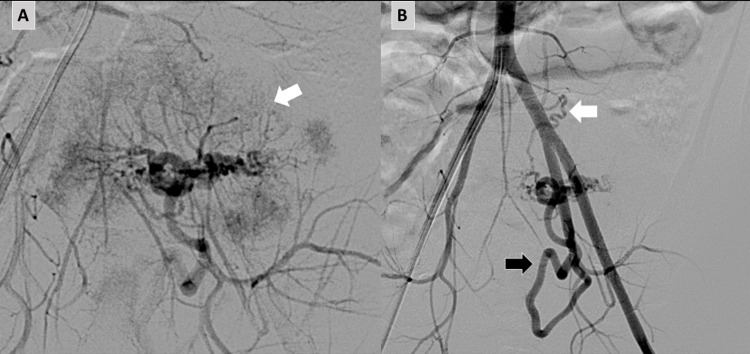
Abdominal arteriography. Arterial blush irrigating the tumor (arrow in A) originated in branches of the internal iliac artery (black arrow in B) and the left gonadal artery (white arrow in B).

Alpha-fetoprotein (AFP) and human chorionic gonadotropin (B-HCG) were normal. Prior to surgical resection, abdominal arteriography was performed with selective embolization and occlusion of the vessels. Two vascular blushes originating in branches of the internal iliac artery and the left gonadal artery were observed irrigating the tumor (Figure 3).

**Figure 4:**
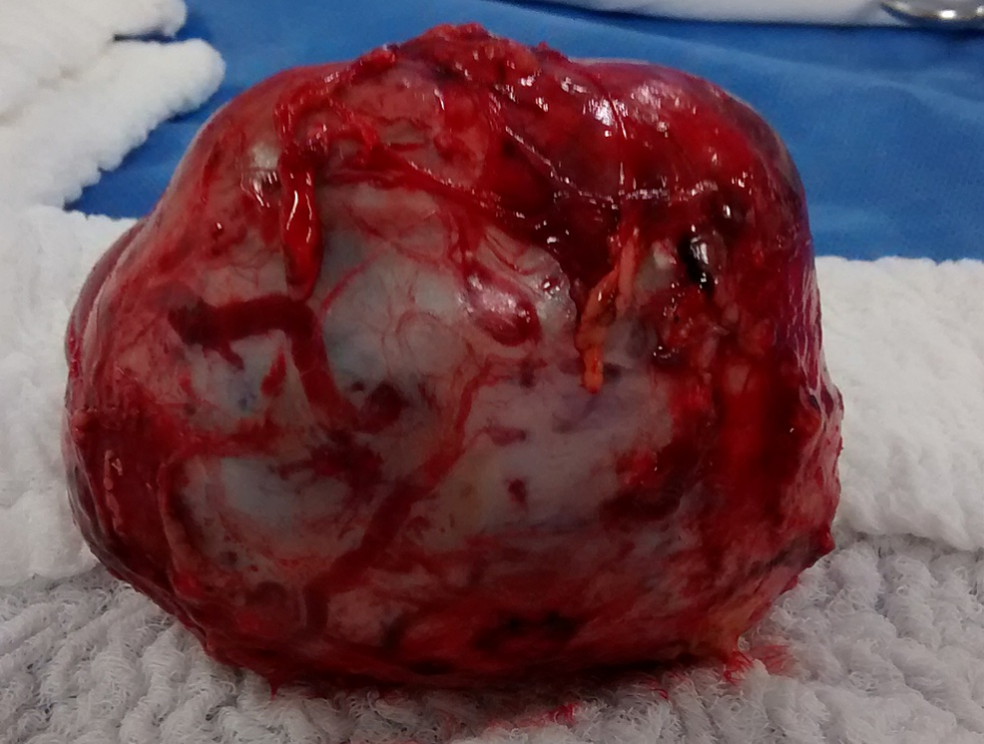
Gross appearance of the specimen. Left ovarian mass measuring 5 x 2.5 x 3 cm, with significant vascularization and involvement of the entire stroma.

**Figure 5:**
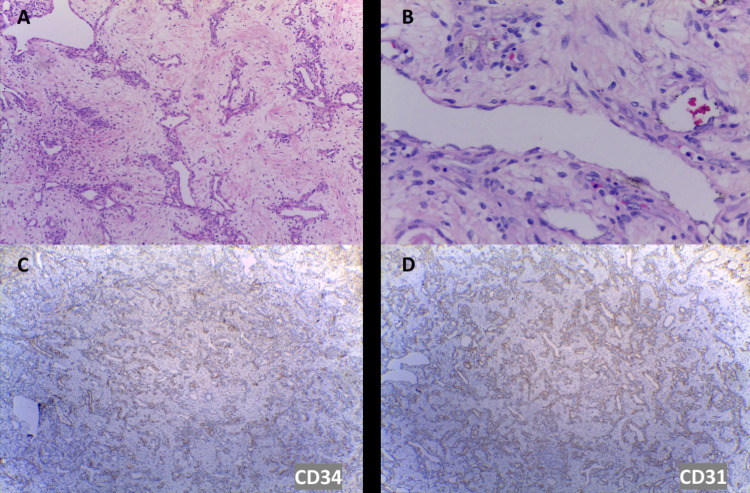
Histopathology images. Hematoxylin and eosin (H&E) staining (A and B) and immunohistochemical (IHC) staining for CD31 and CD34 (C and D). Ovarian tumor with multiple, well-shaped, vascular spaces, occupied by erythrocytes. No severe cellular atypia or necrosis was observed. Positivity for CD31 and CD34 was observed.

A left salpingo-oophorectomy was performed with the removal of a solid mass (Figure 4). The pathological anatomy showed an ovarian neoplasm made up of oval cells with vesicular chromatin, inconspicuous nucleoli, and eosinophilic cytoplasm grouped into cords, and multiple, well-shaped vascular spaces, occupied by erythrocytes. On immunohistochemical staining, tumor cells were positive for CD34, CD31, and factor VIII. These findings confirmed the diagnosis of EHE of the left ovary (Figure 5).

## Discussion

EHE is a rare vascular tumor, originating from endothelial or pre-endothelial vascular cells [1] and mainly affecting young women [2]. EHE was first described in 1975 by Dail and Liebow as an aggressive bronchoalveolar carcinoma called an intravascular bronchoalveolar tumor, later recognized as pulmonary EHE [6]. EHE represents less than 1% of vascular tumors [1]. It is a well-differentiated tumor with a low degree of malignancy, generally diagnosed in adults between the second and third decades of life, although it can be diagnosed in children [1,3,5]. This tumor is more prevalent in the female population, where liver and lung presentations are common [3,5]. The estimated prevalence is less than one in 1,000,000 [1,3,5].

Its involvement is more frequent in the lungs, liver, bones, and soft tissues, although it can occur in any tissue, and in 10% of cases, it can be multifocal. Involvement of the female genital organs, such as the clitoris, vulva, and ovaries, has been described in adult case reports, but no cases have been reported in the pediatric female genital system [3,5,7]. The clinical course of pediatric EHE is unpredictable due to its low prevalence [5].

The etiology is not yet clear. No clear predisposing factors have been described, although a possible association with trauma, radiation therapy, and hormonal factors has been reported [3]. Another possible association with EHE is chronic infection with Bartonella spp. [1]. Vascular differentiation markers such as FLi-1 and chromosomal translocation t(1;3)(p36.3;q25) encode the calmodulin-binding transcription activator 1 (CAMTA1) gene on chromosome 1 and the WW domain-containing transcription regulator 1 (WWTR1) gene on chromosome 3 and the translocation yes-associated protein 1 (YAP1)/transcription factor E3 (TFE3) have been associated to EHE [1,4]. WWRT1/CAMTA1 translocation was found in 90% of patients and YAP1/TFE3 translocation in 10% of patients [5]. EHE has overexpression of vascular endothelial growth factor (VEGF) [5].

The clinical and histological characteristics of EHE are intermediate between benign vascular tumors such as hemangiomas and malignant tumors such as angiosarcomas, although it is part of the malignant tumors according to the 2018 International Society for the Study of Vascular Anomalies (ISSVA) classification [3,8].

The definitive diagnosis is made with immunohistochemistry. The tumor is made up of endothelial cells with minimal nuclear pleomorphism, arranged in cords, short strands, or small nests, with minimal vascular differentiation. In the most aggressive cases, there is increased mitotic activity, significant atypia, focal cell excision, and necrosis. On immunohistochemical staining, tumor cells are positive for at least one vascular marker: CD31, CD34, factor VIII, vimentin, and Ulex europaeus agglutinin 1 (UEA-1); and most cases are negative for cytokeratin (3,7). CD31 has a sensitivity of 100% and a specificity of 62% for the diagnosis of EHE [3].

The characteristics of ovarian EHE images are not well defined due to their rarity; only one case has been previously described in the literature of an adult woman with ultrasound findings of a solid tumor with heterogeneous echogenicity and venous and arterial vascularization in color Doppler mode. No previous description of computed tomography, magnetic resonance imaging, or arteriographic features has been performed [7].

To date, ovarian EHE has not been described in the pediatric population. Due to the lack of knowledge of the characteristics of the image, the diagnosis is currently made by histological confirmation and immunohistochemical staining. However, in the presence of a solid adnexal hypervascular tumor, it is important to think of EHE as a possible differential diagnosis.

The differential diagnosis includes multiple ovarian tumors such as hemangiomas, teratomas, dysgerminomas, choriocarcinomas, sex cord-stromal tumors, and superficial stromal epithelial tumors. Tumor markers and the age of the patient are very helpful in making a proper differential diagnosis [9]. Mature cystic teratomas are diagnosed on ultrasound due to echogenic sebaceous material and calcifications, and on MRI, the sebaceous component can be identified with fat saturation sequences [9,10]. According to tumor markers, AFP may be elevated in immature teratomas and the Sertoli-Leydig cell tumor group of the ovary, with the latter being the most virilizing tumor [10-12]. B-HCG is elevated in dysgerminomas and choriocarcinomas [9,13]. Lactic dehydrogenase (LDH) is elevated in dysgerminomas and may be elevated in choriocarcinoma and immature teratomas, and serum cancer antigen (CA)-125 is elevated in epithelial-stromal tumors [14].

EHE is usually indolent; however, there is a risk of recurrence or metastasis. In some cases, EHE remains stable for many years with further progression or has an aggressive clinical course from diagnosis [1,3,5]. Overall five-year survival is 73-81%, but in high-risk patients, it is reduced to 59% [1,4,5].

## Conclusions

EHE is a rare and low prevalence tumor. This is the first report of ovarian EHE in a pediatric patient. Imaging findings are poorly understood due to the rarity of the disease but it should be a differential diagnosis in patients with hypervascular ovarian tumors. Diagnostic images are essential for the characterization and stratification of the lesion. In this patient, arterial embolization prior to surgical resection decreased the risk of bleeding, facilitating the procedure and reducing complications.
